# Exosome-derived proteins in gastric cancer progression, drug resistance, and immune response

**DOI:** 10.1186/s11658-024-00676-5

**Published:** 2024-12-24

**Authors:** Jiayu Wang, Huan Zhang, Juntao Li, Xiangyu Ni, Wenying Yan, Yueqiu Chen, Tongguo Shi

**Affiliations:** 1 Department of Cardiovascular Surgery of The First Affiliated Hospital and Institute for Cardiovascular Science, Suzhou Medical College of Soochow University, Soochow University, Suzhou, 215007 China; 2 Jiangsu Institute of Clinical Immunology, The First Affiliated Hospital of Soochow University, 178 East Ganjiang Road, Suzhou, 215000 China; 3 Department of Gastroenterology, The First Affiliated Hospital of Soochow University, Suzhou, China; 4 Department of Bioinformatics, School of Biology and Basic Medical Sciences, Suzhou Medical College of Soochow University, 199 Renai Road, Suzhou, 215123 China; 5 Center for Systems Biology, Soochow University, Suzhou, China; 6 Jiangsu Province Engineering Research Center of Precision Diagnostics and Therapeutics Development, Suzhou, China

**Keywords:** Gastric cancer, Exosome, Exosomal protein, Biomarker, Drug resistance, Immune response, Therapy

## Abstract

Gastric cancer (GC) represents a prevalent malignancy globally, often diagnosed at advanced stages owing to subtle early symptoms, resulting in a poor prognosis. Exosomes are extracellular nano-sized vesicles and are secreted by various cells. Mounting evidence indicates that exosomes contain a wide range of molecules, such as DNA, RNA, lipids, and proteins, and play crucial roles in multiple cancers including GC. Recently, with the rapid development of mass spectrometry-based detection technology, researchers have paid increasing attention to exosomal cargo proteins. In this review, we discussed the origin of exosomes and the diagnostic and prognostic roles of exosomal proteins in GC. Moreover, we summarized the biological functions of exosomal proteins in GC processes, such as proliferation, metastasis, drug resistance, stemness, immune response, angiogenesis, and traditional Chinese medicine therapy. In summary, this review synthesizes current advancements in exosomal proteins associated with GC, offering insights that could pave the way for novel diagnostic and therapeutic strategies for GC in the foreseeable future.

## Background

According to global cancer statistics in 2020, gastric cancer (GC) ranks fifth in terms of its prevalence rate and fourth as the leading cause of global cancer-related mortality [1]. Multiple risk factors, such as genetics (genetic susceptibility, epigenetics), *Helicobacter pylori*, intestinal microbes, and chronic inflammation, contribute to the development and progression of GC [1, 2]. Conventional therapeutic modalities, such as endoscopic resection, surgery, chemotherapy, radiotherapy, and targeted therapy, have significantly improved the prognosis of patients with GC. However, the survival rate of patients diagnosed at advanced stage or presenting with metastasis remains notably low [3, 4]. Consequently, the identification of novel, effective diagnostic biomarkers and therapeutic strategies assumes paramount importance in ameliorating the prognosis and enhancing the quality of life for individuals afflicted with GC.

Exosomes, also termed intraluminal vesicles (ILVs), are extracellular nano-sized vesicles that can be secreted by various cells such as immune cells, fibroblasts, endothelial cells, and tumor cells, facilitating the transfer of cellular molecular constituents, including proteins, DNA, lipids, glycoconjugates, and nucleic acids, to recipient cells [5–7]. It has been demonstrated that exosomes play crucial roles in intercellular communication, cell growth, metastasis, survival, immune escape, and drug resistance [8, 9]. In recent years, with the development of mass spectrometry-based detection technology and exosome research, researchers have paid increasing attention to exosomal cargo proteins. Exosomal protein can be extracted from many types of body fluid and can be effective biomarkers for disease diagnosis [10, 11]. Moreover, proteins derived from exosomes exert significant influence on crucial processes, such as proliferation, metastasis, drug resistance, and evasion of immune surveillance, across multiple types of cancers [12–14].

In this review, we center on elucidating the diagnostic and prognostic significance of exosomal proteins while summarizing their involvement and underlying molecular mechanisms in modulating the development and therapeutic responses of GC. These insights aim to foster the advancement of novel diagnostic and therapeutic strategies for GC rooted in exosomal proteins.

### Exosome biogenesis

Mounting evidence has indicated that exosomes frequently harbor specific proteins, often utilized as markers for exosome characterization. Tetraspanin proteins, such as CD63 and CD81, heat shock protein 70 (HSP70), lipidanchored proteins including CD39, CD73, CD55, CD59, and Glypcian-1, along with tumor susceptibility gene 101 protein (TSG101), are commonly employed as markers for exosomes [8, 15–18]. Nonetheless, owing to the lack of standard extraction methods, these biomarkers for exosomes remain elusive and need to be verified by further proteomic studies.

**Fig. 1:**
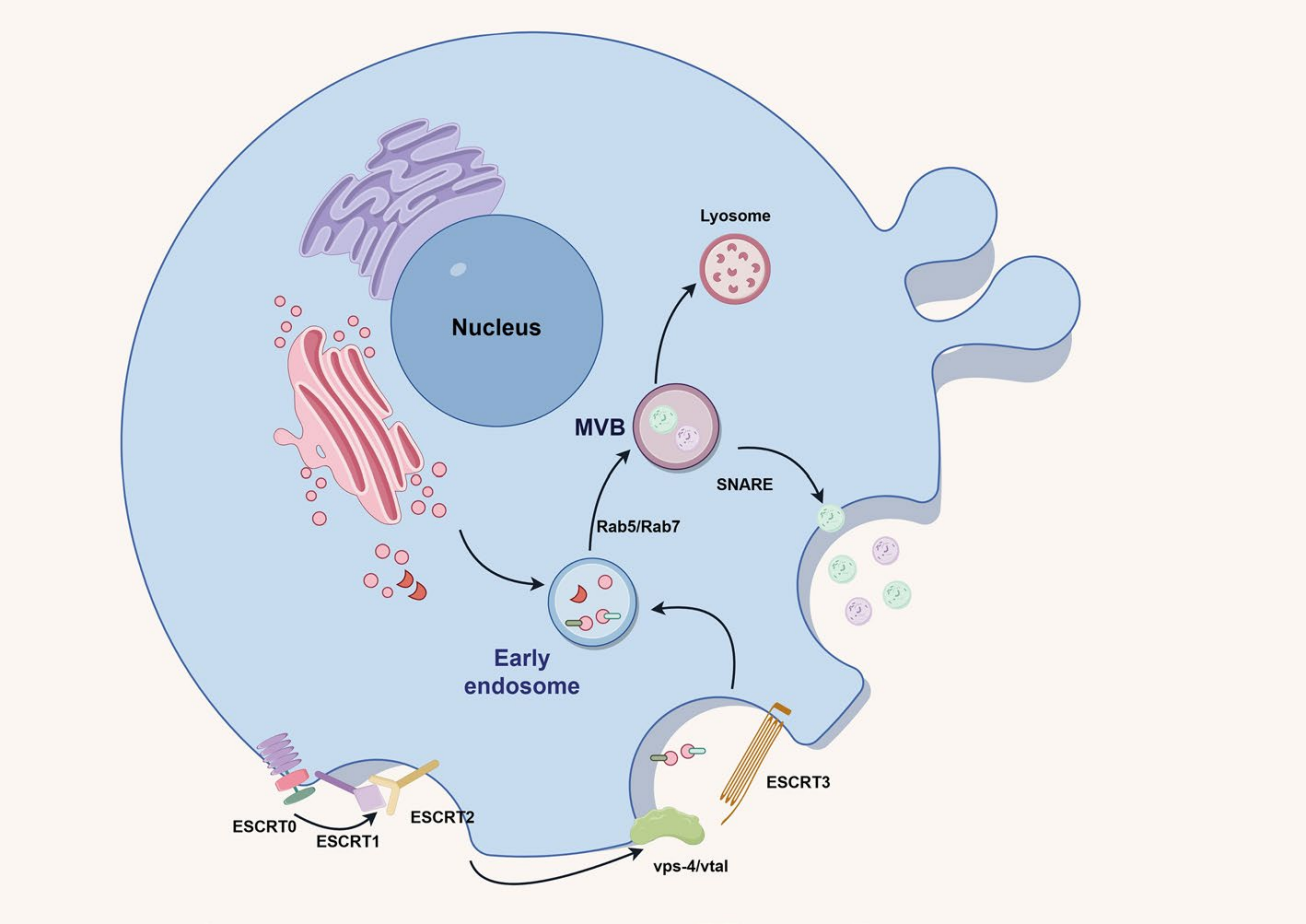
The biogenesis of exosomes in ESCRT-dependent mechanisms. The graphic was created by Figdraw (www.figdraw.com)

The precise mechanism of exosome biogenesis remains still unclear, multiple mechanisms, such as the endosomal sorting complex required for transport (ESCRT)‑dependent pathway, the Alix‑dependent pathway, and the ESCRT‑independent pathway, have been demonstrated to be involved in the formation of exosomes [19]. Among these, the ESCRT‑dependent pathway-mediated mechanism is a classic pathway (Fig. 1) [19, 20]. Four subcomplexes (ESCRT-0, -I, -II, -III) constitute ESCRT, which cooperates with ATPase vacuolar protein sorting 4 homolog A (VPS4) in a stepwise manner. In brief, ESCRT-0 recognizes mono- or poly-ubiquitylated cargo proteins and promotes the initiation of exosome budding. After ESCRT-0 recruits ESCRT-I and ESCRT-II complexes, an ESCRT-cargo-enriched zone is formed, and then the ESCRT-II complex induces the ESCRT-III assembly. Next, ESCRT-III and VPS4 promote vesicle budding by recruiting deubiquitination machinery and packaging cargo into maturing vesicles [21–23]. Following this, inward buds develop sequentially to form endosomes and multivesicular bodies (MVBs) in small GTPases Rap5- and Rap7-dependent manner, MVBs are degraded in lysosomes or fuse with the cell membrane to release contents, such as exosomes [6, 24, 25].

### Exosome‑derived proteins participate in GC progression, drug resistance, and immune response

#### Exosomal cargo proteins regulate GC cell proliferation and metastasis

The uncontrolled proliferation and heightened migratory ability of cancer cells are the major cause of treatment failure for patients with cancer. Identifying specific targets or molecular pathways involved in malignant behavior of cancer cells may pave the road to effective cancer therapy [26–28]. An expanding body of evidence has shown that exosome-derived proteins involved in GC cell proliferation, migration, and invasion have become potential targets for pharmacological or genetic interventions of GC (Table 1).

**Table 1:** The roles of exosome cargo proteins in GC

Origin of exosomes	Proteins	Target cells	Response in target cells	Molecular mechanism	Refs.
MSC	UBR2	MFC cell	Proliferation, migration, stemness	Wnt/β-Catenin	[29]
GC cell	MET	Macrophage	IL-1β production	NA	[31]
TAM	ApoE	GC cell	Cytoskeleton, migration	PI3K/Akt	[32]
GC cell	TRIM3	GC cell	Proliferation, migration	Regulating stem cell factors and EMT regulator	[44]
HFE-145	GKN1	GC cell	EMT, migration, invasion	HRas/Raf/MEK/ERK	[45]
GC cell	NNMT	HMrSV5 cell	Peritoneal metastasis	TGF-β/smad2	[34]
GC cell	CLIC1	GC cell	Vincristine resistance	P-gp and Bcl-2	[50]
GC cell	RPS3	GC cell	Cisplatin resistance	PI3K/Akt/cofilin-1	[53]
GC cell	PD-L1	T cell	5-FU resistance	Induced T cell apoptosis and inhibited T cell activation	[56]
GC cell	GRP78	GC cell	Stemness	NA	[95]
GC cell	HMGB1	Neutrophil	Autophagy, protumor activation	TLR4/NF-κB	[69]
GC cell	PD-L1	T cell	Decreased CD69 and PD-1 expression	Stable and MHC-I expression	[71]
GC cell	PD‑L1	T cell	Activation and proliferation	LSD1	[72]
GC cell	TGF-β1	T cell	Differentiation of CD25 + CTLA4 + FOXP3 + Tregs	NA	[74]
GC cell	THBS1	γδ T cell	Cytotoxicity	m6A methylation/RIG-I-like signaling pathway	[77]
GC cell	GRP78	Vascular endothelial cell	Angiogenesis	AKT	[40]
GC cell	PD-L1	Bone marrow cells	Expansion and differentiation of MDSC	NA	[93]
GC cell	PKM2	Macrophages	Differentiation of TAM	NA	[94]

*MSC* mesenchymal stem cell, *UBR2* ubiquitin-protein ligase E3 component n-recognin 2, *MFC* murine foregastric carcinoma, *GC* gastric cancer, *MET* mesenchymal–epithelial transition factor, *TAM* tumor-associated macrophage, *ApoE* apolipoprotein E, *TRIM3* tripartite motif-containing 3, *EMT* epithelial–mesenchymal transition, *GKN1* Gastrokine 1, *HFE-145* immortalized gastric epithelial cells, *NNMT* nicotinamide N-methyltransferase, *HMrSV5 cell* an established human peritoneal mesothelial cell line, *CLIC1* chloride intracellular channel 1, *P-gp* P-glycoprotein, *RPS3* ribosomal protein S3, *5-FU* 5-fluorouracil, *HMGB1* high mobility group box 1, *LSD1* lysine‑specific demethylase 1, *THBS1* thrombospondin 1, *GRP78* glucose-regulated protein 78, *PKM2* pyruvate kinase M2, *NA* not applicable, *MDSC* myeloid-derived suppressor cell

Certainly, several exosomal cargo proteins have essential roles in promoting GC cell proliferation, migration, and invasion. For instance, ubiquitin-protein ligase E3 component n-recognin 2 (UBR2) was enriched in exosomes secreted by p53-deficient mouse bone marrow mesenchymal stem cells (MSCs). Upon delivery into murine foregastric carcinoma (MFC) cells, UBR2 heightened cell proliferation, migration, and the expression of stemness-related genes (*Nanog*, *OCT4*, and *LIN28*). These effects were mediated through the Wnt/β-Catenin pathway [29]. Moreover, *Helicobacter pylori* (*H*. *pylori*) infection has been reported to trigger chronic inflammation that has been associated with GC [30]. Che et al. observed that *H*. *pylori* infection induced a time-dependent increase in cell-surface receptor tyrosine kinase mesenchymal–epithelial transition factor (MET) expression in the GC cell exosomes, and the exosomal MET-educated macrophages promoted tumor growth and progression in vitro and in vivo in an IL-1β-dependent manner [31]. A study of apolipoprotein E (ApoE) showed that tumor-associated macrophage (TAM)-derived exosomal ApoE prompted the activation of the PI3K/Akt signaling pathway within recipient GC cells. This activation led to a remodeling of the cytoskeleton in GC cells, thereby promoting their migratory capabilities [32]. Peritoneal carcinomatosis, a prominent form of metastatic dissemination, stands as a leading cause of recurrence among patients with GC [33]. Zhu et al. showed that GC cell-derived exosomal nicotinamide N-methyltransferase (NNMT), an S-adenosyl-L-methionine-dependent cytoplasmic enzyme, promoted the malignancy of HMrSV5 cells, an established human peritoneal mesothelial cell line, by activating TGF-β/Smad2 signaling [34]. Angiogenesis and lymphangiogenesis hold crucial roles in tumor growth and metastasis in multiple cancers including GC [35]. Many factors (e.g., exosomes) have been reported to affect angiogenesis and lymphangiogenesis in the context of various cancers [36, 37]. Glucose-regulated protein 78 (GRP78) is a member of the heat shock protein 70 superfamily, which serves as an important regulator in many diseases [38, 39]. A study by Kanako Iha et al. indicated that GRP78-containing exosomes significantly increased the proliferation rate, migration capacity, and tube formation of endothelial cells through the AKT pathway [40]. These findings underscore the multifaceted influence of exosomal cargo proteins, such as UBR2, NNMT, and GRP78, in modulating key pathways related to proliferation, peritoneal metastasis, and angiogenesis, thus contributing to the intricate landscape of GC progression.

On the contrary, various exosomal cargo proteins demonstrate an inhibitory impact on the proliferation and metastasis of gastric cancer (GC) cells. One such example is tripartite motif-containing 3 (TRIM3), a member of the TRIM protein family categorized within the RING-type E3 ubiquitin ligase subfamilies, known for its tumor-suppressive role in multiple cancers [41–43]. Fu et al. conducted a study where they analyzed the proteomic profile of exosomes isolated from the serum of patients with GC using liquid chromatography tandem mass spectrometry (LC–MS/MS). Their findings revealed a reduction in exosomal TRIM3 levels in the serum of patients with GC. Functionally, decreased levels of exosomal TRIM3 were associated with suppressed proliferation and migration of MGC-803 and SGC-7901 cells in vitro. Furthermore, in vivo experiments demonstrated that exosomal TRIM3 inhibited the growth and metastasis of MGC-803 cells [44]. Gastrokine 1 (GKN1), a stomach-specific protein, is produced by gastric mucosal epithelium and is able to be secreted into extracellular space as an exosomal cargo protein. Then, exosomal GKN1 from HFE-145 cells (an immortalized gastric epithelial cell line) curbed GC cell epithelial-mesenchymal transition (EMT), migration, and invasion through the HRas/Raf/MEK/ERK signaling pathways [45].

**Fig. 2:**
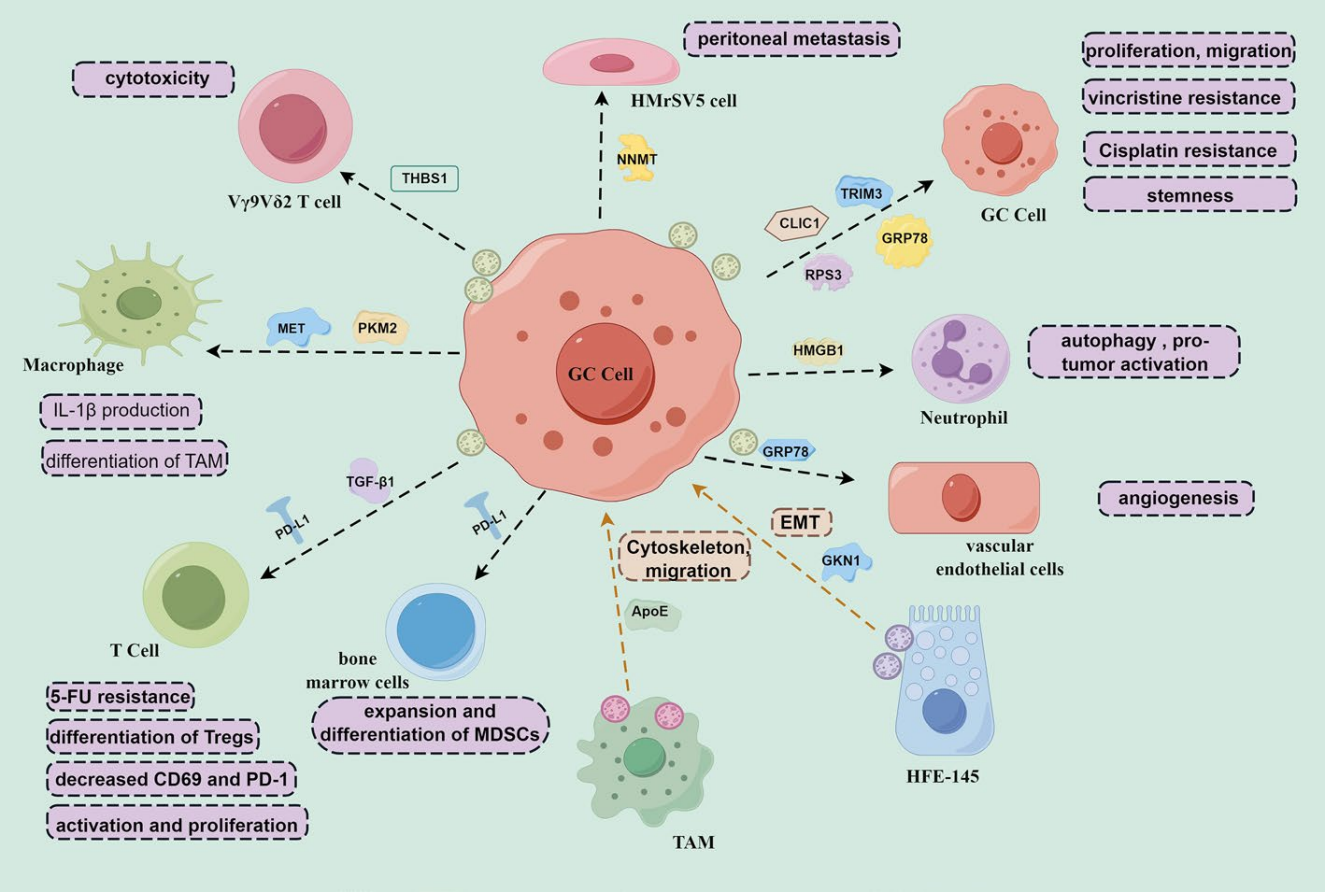
Roles of exosomal proteins in GC. Exosomal cargo proteins are involved in modulating the proliferation, metastasis, drug resistance, stemness, immune response, and angiogenesis in GC. The graphic was created by Figdraw (www.figdraw.com)

In summary, in the context of GC development, exosomal proteins secreted by tumor cells, MSCs, immune cells, and other sources facilitate or suppress the proliferation, migration, and invasion of GC cells through different ways (Fig. 2). These findings indicate that exosomal proteins are beneficial or obstructive for the growth and metastasis in GC. Consequently, these proteins hold promise as potential targets for leveraging exosome-based therapies in the management of GC.

#### Exosomal cargo proteins participate in drug resistance in GC

Currently, chemotherapy is still considered an effective treatment for GC, particularly for advanced GC. However, the emergence of chemotherapy resistance significantly hampers its effectiveness in treating GC [46, 47]. Exosomes, as an important communicator of intercellular communication, participate in drug resistance delivery [9, 48]. Notably, Exosomal cargo proteins play important roles in the transfer of tumor resistance (Fig. 2). One such protein, chloride intracellular channel 1 (CLIC1), a 241-amino acid ion channel protein, has been implicated in various cellular pathophysiological activities and tumor progression, including chemoresistance [49]. The level of exosomal CLIC1 was consistent with that of donor GC cells, and exosomal CLIC1 could induce the development of vincristine resistance in GC by upregulating P-gp and Bcl-2 [50]. Cisplatin (DDP) is widely used as a front-line chemotherapeutic agent for GC, but chemoresistance limits the effectiveness of chemotherapy and leads to treatment failure in the majority of cases [51, 52]. Through LC–MS/MS analysis, ribosomal protein S3 (RPS3) was identified as highly expressed in exosomes derived from DDP-resistant SGC7901 cells. Exosomal RPS3 was observed to enhance chemoresistance in cisplatin-sensitive GC cells via activation of the PI3K-Akt-cofilin-1 signaling pathway [53]. Resistance to 5-fluorouracil (5-FU), another chemotherapy drug, poses a significant challenge in GC treatment [54, 55]. Zhang et al. discovered markedly elevated levels of exosomal PD-L1 in patients with stage III–IV GC after repeated cycles of 5-FU treatment. Notably, the upregulated exosomal PD-L1 was more prominent in clinical 5-FU nonresponders compared with responders. Importantly, exosomal PD-L1 induced apoptosis in Jurkat T cells and inhibited T cell activation in peripheral blood mononuclear cells (PBMCs), signifying its role in impeding the immune response [56]. These findings collectively suggest that exosomal cargo proteins serve as functional signals contributing to acquired chemoresistance in GC (Fig. 2). This underscores the significance of exosomes in transmitting resistance mechanisms and highlights their potential as targets for addressing chemotherapy resistance in GC.

#### Exosomal cargo proteins are involved in the immune response in GC

Cancer immunotherapy has become a powerful clinical strategy for treating various cancers including GC [57–59]. The fundamental mechanism behind cancer immunotherapy involves reinvigorating the antitumor immune response while circumventing pathways that facilitate tumor escape [60, 61]. Exosomes and their cargo proteins have shown potential uses in cancer immunotherapy because of their immunogenicity and molecular transfer functions [62, 63]. Exosomal proteins originating from GC cells or various immune cells exert both immune-stimulatory and immune-suppressive effects within the tumor microenvironment of GC (Fig. 2). These proteins play a multifaceted role in modulating immune responses, thereby influencing the balance between immune activation against the tumor and immune evasion mechanisms within the GC microenvironment.

Neutrophils, a crucial component of the tumor microenvironment, often correlate with immune evasion, tumor growth, and metastasis in various human cancers [64–66]. As a nonhistone protein, high mobility group box 1 (HMGB1) is widely present in the nucleus of mammalian cells, and has been found to play dual roles in tumor immunity, exerting pro- or antitumor immune effects [67, 68]. In a recent study, it was observed that GC cell-derived exosomal HMGB1 triggered autophagy and prompted a protumor activation in neutrophils via the TLR4/NF-κB signaling pathway [69]. This finding sheds light on how exosomal HMGB1 influences neutrophil behavior within the tumor microenvironment, contributing to the protumorigenic processes associated with GC progression.

T cells represent a significant immune cell population within the tumor microenvironment, playing pivotal roles in tumor immunity [70]. It has been reported that exosomal PD-L1 significantly decreased T cell surface CD69 and PD-1 expressions, suggesting that the levels of exosomal PD-L1 reflect the immune status in patients with GC [71]. Shen and colleagues showed that histone lysine‑specific demethylase 1 (LSD1), the first histone demethylase, could enhance the enrichment of PD-L1 in GC-derived exosomes, which further impair T cell response in GC [72]. TGF-β1 is a cytokine, inducing the conversion of naïve T cells into FOXP3+ regulatory T cells [73]. Then, exosomal TGF-β1 expression was positively associated with ratios of FOXP3+ Treg cells in draining LNs. Importantly, GC-derived exosomal TGF-β1 could induce the differentiation of CD25+ CTLA4+ FOXP3+ Tregs from naïve T cells [74]. These observations collectively highlight the influence of exosomal cargo proteins on regulating T cell function and differentiation within the context of GC.

Gamma delta (γδ) T cells, a common type of immune cell, primarily CD4− CD8− double-negative, have been demonstrated to be modulated by exosomes in GC [75, 76]. Recently, our team found that GC cell-derived exosomal thrombospondin 1 (THBS1) markedly enhanced the cytotoxicity of Vγ9Vδ2 T cells in vitro and in vivo. Mechanistically, we elucidated that exosomal THBS1 enhanced the function of Vγ9Vδ2 T cells by activating the RIG-I-like signaling pathway in an m6A methylation-dependent manner, implying that intervening exosomal THBS1 may have important implications for Vγ9Vδ2 T cell-based immunotherapy in the context of treating GC [77].

In short, the interplay between exosomes and the tumor immune response relies on the intricate interactions among various immune cells and tumor cells, fueling significant interest in the field of cancer immunotherapy [78, 79]. This section summarizes the existing studies that delineate the roles of exosomal proteins in regulating neutrophils, T cells, and γδ T cells within the tumor microenvironment of GC. However, current literature is sparse regarding the modulation of immune functions of other immune cell types, such as TAMs, dendritic cells, myeloid-derived suppressor cells and NK cells, by exosomal proteins in GC. Consequently, there exists a considerable need for further investigation and comprehensive understanding of how exosomal proteins influence the tumor’s pro- or antitumor immune responses. Such endeavors would lay the groundwork for developing innovative exosome-based immunotherapy strategies tailored for GC.

### Exosomal cargo proteins act as diagnostic biomarkers and therapeutic targets in GC

#### Exosomal cargo proteins act as new potential GC biomarkers

Several researchers have highlighted the association of exosome-derived proteins with the progression and prognosis of GC, signifying their potential as promising biomarkers for monitoring GC advancement (Table 2). Specifically, the immune checkpoint protein PD-L1, known for its role in interacting with PD-1 and suppressing T cell activation, has been found to exhibit high expression, correlating with poor prognosis in GC [80]. Kabsoo Shin et al. observed that patients with GC who categorized in the low exosomal PD‑L1 group tended to have longer progression-free survival (PFS) compared with those in the high exosomal PD‑L1 group. Furthermore, they noted associations between exosomal PD‑L1 and systemic inflammation markers, immunomodulatory cytokines, and T cells, suggesting that serum-derived exosomal PD‑L1 might reflect the immunosuppressive state in patients with advanced GC [81]. Similarly, Fan et al. conducted a study demonstrating that circulating exosomal PD-L1 predicted poorer survival outcomes in patients with GC and served as an independent prognostic factor in GC [71]. These findings collectively underscore the potential of exosomal PD-L1 as a valuable biomarker in prognosticating and assessing the immunosuppressive status in patients with advanced GC. Frizzled-10 (FZD10), a protein of the Frizzled family, is a cell surface receptor, which is activated by Wnt proteins and involved in the regulation of cellular function [82]. A statistically significant progressive upregulation of exosomal FZD10 level in exosomes and tissues from patients at stage T1 to stages T2–T4 of GC, as compared with healthy subjects. Moreover, there was a strong positive correlation between FZD10 in exosomes and Ki-67 in tumor tissues [83]. Moreover, the plasma exosomal FZD-10 levels in the healthy controls and patients with CRC or GC indicated that its expression in oncological patients was higher than in the control group, while, at the end of the treatment (e.g., surgery), it reached values comparable with the average level of controls [84]. Research has focused on the possible relationships between exosomal cargo proteins and tumor metastasis, to discover novel tumor-specific and sensitive cancer biomarkers. The NNMT contents were much higher in patient exosomes isolated from GC with peritoneal carcinomatosis (PM) than in GC without PM [34]. These findings underscored the potential utility of FZD10 and NNMT as potential biomarkers in GC, shedding light on their significance in disease progression and their prospective roles in diagnostic or therapeutic strategies. Chenfei Zhou et al. utilized Nano-HPLC–MS/MS to analyze exosome proteins derived from peripheral blood samples obtained from 12 patients with advanced GC displaying organ-specific metastasis, including distant lymphatic, hepatic, and peritoneal metastases. Through Gene Ontology (GO) and Kyoto Encyclopedia of Genes and Genomes (KEGG) enrichment analyses, they identified that differentially expressed proteins (DEPs) in exosomes from hepatic metastasis were linked to lipid metabolism, while those in exosomes were associated with regulating actin cytoskeleton and glycolysis/gluconeogenesis. Notably, among these DEPs, exosomal CD14 and integrin linked kinase 1 (ILK1) were found to be correlated with hepatic and peritoneal metastases in patients with GC, respectively [85]. Additionally, serum exosomal CD44 was positively associated with tumor burden in lymph nodes in patients with GC [86]. Furthermore, Wang et al. showed that Wnt5a content in serum exosomes is positively correlated with lymph node metastasis in GC [87]. The expression levels of TGF-β1 in plasma exosomes of patients with GC were also positively correlated with tumor node metastasis (TNM) stage and lymph node metastasis [74]. These findings collectively suggest that exosomal cargo proteins hold potential indicative value in understanding and predicting GC progression, metastasis, and prognosis.

**Table 2:** Exosome cargo proteins as biomarkers for GC diagnosis

Protein	Biofluid	Detection mode	Progression	Prognosis	Refs.
PD-L1	Serum	ELISA	NA	PFS	[81]
PD-L1	Serum	ELISA	NA	OS	[71]
FZD10	Plasma	Western blotting	TNM stage	NA	[83]
FZD10	Plasma	Western blotting	NA	NA	[84]
NNMT	Peritoneal fluid	Western blotting	Peritoneal carcinomatosis	NA	[34]
CD14	Plasma	Nano-HPLC–MS/MS	Peritoneal metastasis	NA	[85]
ILK1	Plasma	Nano-HPLC–MS/MS	Hepatic metastasis	NA	[85]
CD44	Serum	ELISA	LNM	NA	[86]
Wnt5a	Serum	ELISA	LNM	NA	[87]
TGF-β1	Plasma	ELISA	TNM stage LNM	NA	[74]
HER2	Serum	ELISA	NA	NA	[90]

*ELISA* enzyme-linked immunosorbent assay, *NA* not applicable, *LNM* lymph node metastasis, *TNM* tumor node metastasis, *PFS* progression-free survival, *OS* overall survival, *FZD10* Frizzled-10, *NNMT* nicotinamide N-methyltransferase, *ILK1* integrin linked kinase 1

#### Exosomal cargo proteins are associated with GC-targeted therapy

Targeted therapy for cancer has always been the focus of clinicians. Human epidermal growth factor receptor-2 (HER2)-targeted therapy is one of the most popular targets for translational research in cancer. The overexpression of HER2 is identified in a subset of patients with advanced GC, ranging from 9.0% to 38%, who could potentially benefit from trastuzumab, a humanized monoclonal antibody targeting HER2 [88, 89]. Given the significance of HER2 status in guiding clinical strategies, there is an urgent need to address the heterogeneity of HER2 in GC through innovative methods. Research by Qian Li et al. highlighted that the serum exosomal HER2 level in patients with GC correlated with tissue HER2-positive status. Notably, patients with higher baseline serum exosomal HER2 levels exhibited better outcomes in response to trastuzumab-based therapy [90]. Hence, serum exosomal HER2 emerges as a potential novel biomarker with dual utility: assessing tissue HER2-positive status in GC and offering predictive value for HER2-targeted therapy efficacy. This discovery holds promise in streamlining therapeutic decision-making and optimizing treatment outcomes for patients with GC undergoing HER2-targeted therapy.

#### Exosomal cargo proteins were associated with traditional Chinese medicine therapy in GC

It has widely been believed that traditional Chinese medicine (TCM) is one of the most important alternative and complementary treatment options for GC [91, 92]. Notably, the mechanisms induced by exosomal cargo proteins are closely linked to TCM treatments. For instance, *Jianpi Yangzheng Xiaozheng* decoction (JPYZXZ), an empirical traditional Chinese medicine formula, demonstrated an ability to decrease the expression levels of GC-derived exosomal PD-L1 in MFC murine cells, xenograft GC models, and patients with stage IIA-IIIB GC. This treatment inhibited the transfer of exosomal PD-L1 from GC cells to bone marrow cells, thereby mitigating exosomal PD-L1-induced differentiation and expansion of myeloid-derived suppressor cells (MDSCs) within the tumor microenvironment [93]. Similarly, modified *Jianpi Yangzheng* decoction (mJPYZ), another empirical TCM decoction, exhibited significant potential in prolonging the survival of patients with advanced-stage GC. mJPYZ treatment reduced the abundance of serum exosome pyruvate kinase M2 (PKM2) in patients with advanced GC and xenograft tumor models. Notably, mJPYZ intervened in the transfer of exosomal PKM2 from GC cells to macrophages, thereby mitigating exosomal PKM2-induced differentiation of TAMs within the tumor microenvironment, ultimately impeding the progression of GC [94]. These compelling findings collectively present a rationale for the potential application of traditional Chinese medicine in suppressing GC via mechanisms involving exosomal cargo proteins.

## Conclusions

The rapid advancements in mass spectrometry-based detection technology and exosome research have brought significant attention to the biological functions of exosome cargo proteins in GC. These proteins play a pivotal role in mediating communication between GC cells and diverse cell types, such as MSCs and various immune cells. They actively modulate critical aspects of GC, including cell proliferation, metastasis, drug resistance, and immune response. Moreover, serum exosomal proteins exhibit potential in contributing to GC detection, prognostication, and treatment monitoring. Notably, specific exosomal proteins, such as PD-L1 and PKM2, are linked to the outcomes of traditional Chinese medicine therapy in GC. Nevertheless, despite the strides made, there are still plenty of unknowns in the field of exosomal cargo proteins that deserve to be further explored. Large-scale clinical studies are necessary to validate the relationships between the expression of these biomarkers and the clinicopathological characteristics of patients with GC. Most experiments concerning exosomal proteins have been conducted in vitro and in vivo animal models, leaving questions about the safety, specificity, and efficacy of this strategy in clinical trials. Future research endeavors should aim to address these gaps. Nevertheless, therapies based on exosomal proteins, encompassing targeted therapy, immunotherapy, and traditional Chinese medicine therapy, hold promise in enhancing the prognosis of patients with GC. Continued investigations into exosomal proteins and their therapeutic applications could potentially revolutionize GC treatment, offering improved outcomes for patients in the future.

## Data Availability

Not applicable.

## Abbreviations

GC: Gastric cancer
ILVs: Intraluminal vesicles
HSP70: Heat shock protein 70
TSG101: Tumor susceptibility gene 101 protein
ESCRT: Endosomal sorting complex required for transport
VPS4: Vacuolar protein sorting 4 homolog A
MVBs: Multivesicular bodies
UBR2: Ubiquitin-protein ligase E3 component n-recognin 2
MSCs: Mesenchymal stem cells
H. pylori: Helicobacter pylori
MET: Mesenchymal-epithelial transition factor
ApoE: Apolipoprotein E
TAM: Tumor-associated macrophage
NNMT: Nicotinamide N-methyltransferase
GRP78: Glucose-regulated protein 78
TRIM3: Tripartite motif-containing 3
LC–MS/MS: Liquid chromatography tandem mass spectrometry
GKN1: Gastrokine 1
EMT: Epithelial-mesenchymal transition
CLIC1: Chloride intracellular channel 1
DDP: Cisplatin
RPS3: Ribosomal protein S3
5-FU: 5-Fluorouracil
PBMCs: Peripheral blood mononuclear cells
HMGB1: High mobility group box 1
LSD1: Lysine‑specific demethylase 1
Γδ: Gamma delta
THBS1: Thrombospondin 1
PFS: Progression-free survival
FZD10: Frizzled-10
PM: Peritoneal carcinomatosis
GO: Gene Ontology
KEGG: Kyoto Encyclopedia of Genes and Genomes
DEPs: Differentially expressed proteins
ILK1: Integrin linked kinase 1
PKM2: Pyruvate kinase M2
HER2: Human epidermal growth factor receptor-2
JPYZXZ: Jianpi Yangzheng Xiaozheng decoction
mJPYZ: Modified Jianpi Yangzheng decoction
